# 3-(1*H*-Tetra­zol-5-yl)benzoic acid

**DOI:** 10.1107/S1600536808041482

**Published:** 2008-12-10

**Authors:** Lin Cheng, Ya-Wen Zhang, Jian-Quan Wang, Gong Zhang

**Affiliations:** a Department of Chemistry and Chemical Engineering, Southeast University, Nanjing, 211189, People’s Republic of China; b Department of Chemistry and Chemical Engineering, State Key Laboratory of Coordination Chemistry, Nanjing University, Nanjing, 211189, People’s Republic of China

## Abstract

The title compound, C_8_H_6_N_4_O_2_, is a difunctional compound with a carboxyl­ate and a tetra­zole residue. In the crystal structure, mol­ecules are linked into two-dimensional sheets by inter­molecular N—H⋯O and O—H⋯N hydrogen bonds.

## Related literature

For the applications of tetra­zoles, see: Chen & Tong (2007 ▶); Demko & Sharpless (2001 ▶). For related structures, see: Rizk *et al.* (2005 ▶).
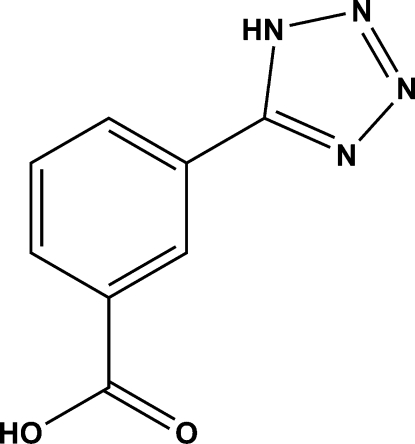


## Experimental

### 

#### Crystal data

C_8_H_6_N_4_O_2_
*M* *_r_* = 190.17
Monoclinic, 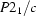

*a* = 5.2501 (10) Å
*b* = 16.805 (3) Å
*c* = 9.3290 (18) Å
β = 99.188 (3)°
*V* = 812.5 (3) Å^3^
*Z* = 4
Mo *K*α radiation
μ = 0.12 mm^−1^
*T* = 293 (2) K
0.45 × 0.14 × 0.13 mm

#### Data collection

Bruker SMART APEX CCD diffractometer
Absorption correction: multi-scan *SADABS* (Sheldrick, 2000 ▶) *T*_min_ = 0.949, *T*_max_ = 0.985
5991 measured reflections
1583 independent reflections
1425 reflections with *I* > 2σ(*I*)
*R*_int_ = 0.018

#### Refinement

*R*[*F* ^2^ > 2σ(*F* ^2^)] = 0.040
*wR*(*F* ^2^) = 0.106
*S* = 1.07
1583 reflections
136 parameters
H atoms treated by a mixture of independent and constrained refinement
Δρ_max_ = 0.19 e Å^−3^
Δρ_min_ = −0.24 e Å^−3^

### 

Data collection: *SMART* (Bruker, 2000 ▶); cell refinement: *SAINT* (Bruker, 2000 ▶); data reduction: *SAINT*; program(s) used to solve structure: *SHELXS97* (Sheldrick, 2008 ▶); program(s) used to refine structure: *SHELXL97* (Sheldrick, 2008 ▶); molecular graphics: *SHELXTL* (Sheldrick, 2008 ▶); software used to prepare material for publication: *SHELXL97*.

## Supplementary Material

Crystal structure: contains datablocks I, global. DOI: 10.1107/S1600536808041482/bt2829sup1.cif

Structure factors: contains datablocks I. DOI: 10.1107/S1600536808041482/bt2829Isup2.hkl

Additional supplementary materials:  crystallographic information; 3D view; checkCIF report

## Figures and Tables

**Table 1:** Hydrogen-bond geometry (Å, °)

*D*—H⋯*A*	*D*—H	H⋯*A*	*D*⋯*A*	*D*—H⋯*A*
O2—H2*B*⋯N4^i^	0.93 (2)	1.76 (2)	2.6664 (15)	164 (2)
N1—H1*A*⋯O1^ii^	0.94 (2)	1.77 (2)	2.7118 (16)	179.1 (19)

Symmetry codes: (i) 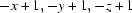; (ii) 
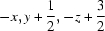
.

**Fig. 1.**
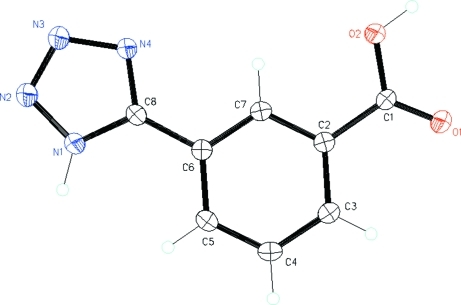
Structure of the title compound with 30% displacement ellipsoids.

**Fig. 2.**
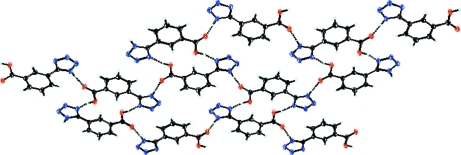
The two-dimensional hydrogen bonding network of the title compound.

**Fig. 3.**
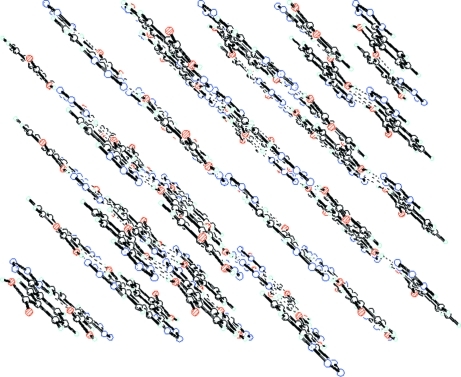
Packing of the title compound with view onto the ac plane.

## supplementary crystallographic information

### Comment

Tetrazoles have been extensively investigated in organic synthetic chemistry for several decades due to the fact that they have wide ranging applications in pharmaceuticals, especially explosives, photography, information recording systems, agriculture, and as precursors to a variety of heterocycles (Chen *et al.* 2007; Demko *et al.* 2001). They have also been used as a type of important multidentate ligands in coordination chemistry. Here, we report the crystal structure of a new tetrazole, 3-(1*H*-tetrazol-5-yl)benzoic acid.

The title compound, C_8_H_6_N_4_O_2_, is a difunctional compound with carboxylate and tetrazole groups. The C=O distance of the carboxylate is 1.216 (2) Å, which is much shorter than the C—O distance of 1.311 (2) Å. In the tetrazole group, the N=N distance is 1.288 (2) Å, and the N—N distances are 1.343 (2) and 1.358 (2) Å, respectively. The C—N distance is 1.333 (2) Å, being close to the C=N distance of 1.325 (2) Å, which is considered to have part double-bond character. In the crystalline state, the molecules are linked to two-dimensional hydrogen-bonding networks by intermolecular N—H···O and O—H···N hydrogen bonds. The N···O distance is 2.712 (2) Å, and the O···N distance is 2.666 (2) Å.

### Experimental

A mixture of 3-cyanobenzoic acid (0.147 g, 1.0 mmol), Cd(NO_3_)_2_.6H_2_O (0.345 g, 1 mmol) and water (8 ml) was was heated in a 15-ml Teflon-lined autoclave at 160 ° for 3 days, followed by slow cooling (5 ° h-1) to room temperature. The resulting mixture was washed with water  and collected. Then, the obtained solids were put into 20 ml water, and 10% Na_2_S aqueous solution was droped to the suspension liquid until that no precipitation appeared. The solution was filtered and the filtrate was acidified with 50% HCl solution until the pH value was 1.0. White products were filtered, washed with water, then dried and collected in 76.2% yield (0.145 g) based on 3-cyanobenzoic acid. Colorless block shaped crystals were collected from the filtrate after the second filtration.

### Refinement

H atoms bonded to N and O atoms were located in a difference map and were freely refined. Other H atoms were positioned geometrically and refined using a riding model with C—H = 0.93 Å and with *U*_iso_(H) = 1.2.

### Crystal data

**Table:**

C_8_H_6_N_4_O_2_	*F*(000) = 392
*M**_r_* = 190.17	*D*_x_ = 1.555 Mg m^−^^3^
Monoclinic, *P*2_1_/*c*	Mo *K*α radiation, λ = 0.71073 Å
Hall symbol: -P 2ybc	Cell parameters from 785 reflections
*a* = 5.2501 (10) Å	θ = 2.4–28.0°
*b* = 16.805 (3) Å	µ = 0.12 mm^−^^1^
*c* = 9.3290 (18) Å	*T* = 293 K
β = 99.188 (3)°	Block, colorless
*V* = 812.5 (3) Å^3^	0.45 × 0.14 × 0.13 mm
*Z* = 4	

### Data collection

**Table:**

Bruker APX CCD diffractometer	1583 independent reflections
Radiation source: fine-focus sealed tube	1425 reflections with *I* > 2σ(*I*)
graphite	*R*_int_ = 0.018
phi and ω scan	θ_max_ = 26.0°, θ_min_ = 2.4°
Absorption correction: multi-scan *SADABS* (Sheldrick, 2000)	*h* = −6→6
*T*_min_ = 0.949, *T*_max_ = 0.985	*k* = −20→20
5991 measured reflections	*l* = −11→11

### Refinement

**Table:**

Refinement on *F*^2^	Secondary atom site location: difference Fourier map
Least-squares matrix: full	Hydrogen site location: inferred from neighbouring sites
*R*[*F*^2^ > 2σ(*F*^2^)] = 0.040	H atoms treated by a mixture of independent and constrained refinement
*wR*(*F*^2^) = 0.106	*w* = 1/[σ^2^(*F*_o_^2^) + (0.0592*P*)^2^ + 0.1634*P*] where *P* = (*F*_o_^2^ + 2*F*_c_^2^)/3
*S* = 1.07	(Δ/σ)_max_ < 0.001
1583 reflections	Δρ_max_ = 0.19 e Å^−^^3^
136 parameters	Δρ_min_ = −0.24 e Å^−^^3^
0 restraints	Extinction correction: *SHELXL*, Fc^*^=kFc[1+0.001xFc^2^λ^3^/sin(2θ)]^-1/4^
Primary atom site location: structure-invariant direct methods	Extinction coefficient: 0.018 (3)

### Special details

**Table:**

Geometry. All e.s.d.'s (except the e.s.d. in the dihedral angle between two l.s. planes) are estimated using the full covariance matrix. The cell e.s.d.'s are taken into account individually in the estimation of e.s.d.'s in distances, angles and torsion angles; correlations between e.s.d.'s in cell parameters are only used when they are defined by crystal symmetry. An approximate (isotropic) treatment of cell e.s.d.'s is used for estimating e.s.d.'s involving l.s. planes.
Refinement. Refinement of *F*^2^ against ALL reflections. The weighted *R*-factor *wR* and goodness of fit *S* are based on *F*^2^, conventional *R*-factors *R* are based on *F*, with *F* set to zero for negative *F*^2^. The threshold expression of *F*^2^ > σ(*F*^2^) is used only for calculating *R*-factors(gt) *etc*. and is not relevant to the choice of reflections for refinement. *R*-factors based on *F*^2^ are statistically about twice as large as those based on *F*, and *R*- factors based on ALL data will be even larger.

### Fractional atomic coordinates and isotropic or equivalent isotropic displacement parameters (Å^2^)

**Table:**

	*x*	*y*	*z*	*U*_iso_*/*U*_eq_	
O1	0.0644 (2)	0.36005 (6)	0.68049 (12)	0.0471 (3)	
O2	0.3759 (2)	0.42002 (6)	0.58687 (13)	0.0496 (3)	
H2B	0.410 (4)	0.3707 (14)	0.550 (2)	0.082 (7)*	
C1	0.1794 (3)	0.42040 (8)	0.65768 (15)	0.0358 (3)	
C2	0.1127 (3)	0.50146 (7)	0.70380 (15)	0.0349 (3)	
C3	−0.0705 (3)	0.51184 (8)	0.79498 (16)	0.0403 (4)	
H3A	−0.1499	0.4680	0.8296	0.048*	
C4	−0.1332 (3)	0.58797 (9)	0.83368 (16)	0.0439 (4)	
H4A	−0.2539	0.5950	0.8955	0.053*	
C5	−0.0189 (3)	0.65372 (8)	0.78180 (16)	0.0392 (3)	
H5A	−0.0640	0.7046	0.8081	0.047*	
C6	0.1642 (3)	0.64386 (7)	0.68994 (14)	0.0338 (3)	
C7	0.2301 (2)	0.56732 (8)	0.65297 (14)	0.0356 (3)	
H7A	0.3544	0.5602	0.5934	0.043*	
C8	0.2910 (3)	0.71171 (7)	0.63149 (14)	0.0338 (3)	
N1	0.2597 (2)	0.78846 (7)	0.66034 (13)	0.0402 (3)	
N2	0.4101 (3)	0.83282 (7)	0.58804 (14)	0.0453 (3)	
N3	0.5310 (3)	0.78398 (7)	0.51637 (14)	0.0445 (3)	
N4	0.4608 (2)	0.70804 (7)	0.54077 (13)	0.0394 (3)	
H1A	0.147 (4)	0.8129 (12)	0.716 (2)	0.067 (5)*	

### Atomic displacement parameters (Å^2^)

**Table:**

	*U*^11^	*U*^22^	*U*^33^	*U*^12^	*U*^13^	*U*^23^
O1	0.0573 (7)	0.0296 (5)	0.0618 (7)	−0.0056 (4)	0.0317 (5)	0.0017 (4)
O2	0.0616 (7)	0.0266 (5)	0.0718 (7)	−0.0021 (4)	0.0446 (6)	−0.0037 (5)
C1	0.0416 (7)	0.0290 (7)	0.0408 (7)	−0.0007 (5)	0.0188 (6)	0.0037 (5)
C2	0.0379 (7)	0.0299 (7)	0.0400 (7)	0.0007 (5)	0.0158 (6)	0.0010 (5)
C3	0.0448 (8)	0.0328 (7)	0.0485 (8)	−0.0023 (6)	0.0231 (6)	0.0013 (6)
C4	0.0468 (8)	0.0408 (8)	0.0510 (8)	0.0015 (6)	0.0285 (7)	−0.0021 (6)
C5	0.0434 (8)	0.0305 (7)	0.0474 (8)	0.0036 (5)	0.0188 (6)	−0.0048 (6)
C6	0.0374 (7)	0.0285 (7)	0.0380 (7)	−0.0004 (5)	0.0133 (5)	−0.0002 (5)
C7	0.0394 (7)	0.0311 (7)	0.0406 (7)	0.0002 (5)	0.0195 (6)	0.0003 (5)
C8	0.0383 (7)	0.0266 (6)	0.0388 (7)	0.0026 (5)	0.0131 (5)	−0.0021 (5)
N1	0.0498 (7)	0.0263 (6)	0.0496 (7)	0.0012 (5)	0.0238 (6)	−0.0017 (5)
N2	0.0564 (8)	0.0290 (6)	0.0559 (8)	−0.0022 (5)	0.0257 (6)	0.0001 (5)
N3	0.0537 (7)	0.0293 (6)	0.0562 (8)	−0.0026 (5)	0.0260 (6)	0.0005 (5)
N4	0.0470 (7)	0.0263 (6)	0.0504 (7)	−0.0010 (5)	0.0247 (5)	−0.0010 (5)

### Geometric parameters (Å, °)

**Table:**

O1—C1	1.2163 (16)	C5—H5A	0.9300
O2—C1	1.3112 (16)	C6—C7	1.3898 (18)
O2—H2B	0.93 (2)	C6—C8	1.4684 (18)
C1—C2	1.4871 (18)	C7—H7A	0.9300
C2—C7	1.3868 (18)	C8—N4	1.3254 (17)
C2—C3	1.3927 (19)	C8—N1	1.3331 (17)
C3—C4	1.3833 (19)	N1—N2	1.3430 (16)
C3—H3A	0.9300	N1—H1A	0.94 (2)
C4—C5	1.381 (2)	N2—N3	1.2882 (17)
C4—H4A	0.9300	N3—N4	1.3576 (16)
C5—C6	1.3961 (18)		
			
C1—O2—H2B	114.1 (14)	C7—C6—C5	119.03 (12)
O1—C1—O2	122.47 (12)	C7—C6—C8	118.74 (11)
O1—C1—C2	124.52 (12)	C5—C6—C8	122.22 (12)
O2—C1—C2	113.01 (11)	C2—C7—C6	120.79 (12)
C7—C2—C3	119.79 (12)	C2—C7—H7A	119.6
C7—C2—C1	119.61 (11)	C6—C7—H7A	119.6
C3—C2—C1	120.59 (11)	N4—C8—N1	106.91 (11)
C4—C3—C2	119.44 (12)	N4—C8—C6	126.31 (11)
C4—C3—H3A	120.3	N1—C8—C6	126.77 (12)
C2—C3—H3A	120.3	C8—N1—N2	109.54 (11)
C5—C4—C3	120.91 (12)	C8—N1—H1A	129.9 (12)
C5—C4—H4A	119.5	N2—N1—H1A	120.5 (12)
C3—C4—H4A	119.5	N3—N2—N1	106.56 (11)
C4—C5—C6	120.02 (12)	N2—N3—N4	110.02 (11)
C4—C5—H5A	120.0	C8—N4—N3	106.96 (10)
C6—C5—H5A	120.0		

### Hydrogen-bond geometry (Å, °)

**Table:**

*D*—H···*A*	*D*—H	H···*A*	*D*···*A*	*D*—H···*A*
O2—H2B···N4^i^	0.93 (2)	1.76 (2)	2.6664 (15)	164 (2)
N1—H1A···O1^ii^	0.94 (2)	1.77 (2)	2.7118 (16)	179.1 (19)

Symmetry codes: (i) −*x*+1, −*y*+1, −*z*+1; (ii) −*x*, *y*+1/2, −*z*+3/2.
